# Digital Transformation of Agriculture through the Use of an Interoperable Platform †

**DOI:** 10.3390/s20041153

**Published:** 2020-02-20

**Authors:** Juan Antonio López-Morales, Juan Antonio Martínez, Antonio F. Skarmeta

**Affiliations:** 1 Department of Information and Communications Engineering, Computer Science Faculty, University of Murcia, 30100 Murcia, Spain; skarmeta@um.es; 2 Odin Solutions S.L, Polígono Industrial Oeste C/Perú, 5, 3°, Oficina 12, 30820 Alcantarilla (Murcia), Spain; jamartinez@odins.es

**Keywords:** IoT platform, data model, smart agriculture, irrigation water, precision irrigation, interoperability, NGSI-LD, FIWARE

## Abstract

The continuous evolution of the agricultural sector justifies the incorporation and adaptation of the latest technologies. Nowadays, managing crops is possible through Internet-based technologies. Their application allows for the exploitation of information and the development of isolated applications, which, although powerful, create challenges for obtaining scalable predictions throughout the useful life of farms. To address this problem, a data model was defined to improve the management of crop plots in irrigation communities and simultaneously monitor crop needs. Consequently, the objective of this study was to create an open and interoperable platform based on standard interfaces and protocols to enable the integration of heterogeneous sources of information, while ensuring interoperability with other third-party solutions for exchanging and exploiting such information. Standard and open interfaces and protocols form the basis of the platform, thereby unifying all information in a single data model, which facilitates the better use and dissemination of information. The system was fully instantiated in a real prototype in an irrigation community; the software improved water irrigation management for the farmers connected to the platform.

## 1. Introduction

Society is characterized by uncertainty and constant technological changes. The productive sectors that are more digitized tend to improve their productivity faster than those that are less digitized. Irrigation farming communities have benefited for many years from the use of information and communications technology (ICT), which allows them to improve the current agricultural development, facilitate the fulfillment of daily tasks, and convert farms into efficient and sustainable production systems. The use of these new technologies not only enables the digital transformation process demanded by the sector, but also supports irrigation communities and other farming groups with tools for analysis and prediction, improving optimization.

The digital transformation and continuous technological advances that are currently occurring, e.g., the Internet of Things (IoT) [1], big data, cloud computing [2], artificial intelligence, and aerial images [3] to name a few, are providing the agricultural sector with new tools that help determine the real needs of farms and improve their efficiency. Specifically, the use of the IoT has changed the traditional paradigm regarding access to and the management of sensors and actuators, making all objects accessible through the Internet, and transferring the management and integration of information and real knowledge into the digital world [4]. This transformation has produced improvements compared to the techniques traditionally used [5], allowing new mechanisms to help in the management of farms. These advantages are mainly focused on the following:

- *Problem detection*: The implementation of new technologies such as satellite images, variable application algorithms, drones, high-tech sensors, mobile applications, and GPS guides, allowing for the assessment of crop status and the detection of problems (improper fertilizer use, water stress, changing weather conditions, and pest monitoring) before they start to interfere with crop  performance;
- *Productivity improvement*: The sensorization of crops allows for the establishment of patterns of planting and fertilization depending on factors such as the type of seeds and soil conditions, to improve crop production levels;
- *Improvement in decision making*: By analyzing and monitoring agronomic parameters, crop water needs, and precipitation forecasts, it is possible to identify which areas of land need more water and schedule irrigation to vary the volume of water applied without imposing water stress on the crop;
- *Behaviour analysis*: As a result of continuous crop monitoring and the analysis of the relationships among different elements, such as yield, energy efficiency, and the agricultural practices used, the most beneficial actions and those that must be eliminated or modified can be identified.

Motivated by the digital transformation, the agricultural sector is providing its farms with new devices and services (sensors, actuators, weather information, drones, and satellite images) that allow for the optimization of the resources, to improve productivity and simultaneously reduce the impact on the environment. For the digital transformation of the agricultural sector, a common integration framework is needed that unifies the entire dataset, so that new services are generated according to the needs of the sector. The data generated by the farms are crucial and have to be part of a unique semantic model that organizes and evaluates all the data collected.

At the European level, an open initiative, FIWARE [6], offers a framework using a set of standard and open Application Programming Interfaces (APIs) based on Next Generation Service Interface (NGSI), promoted by the Open Mobile Alliance (OMA), or Next Generation Service Interfaces with Linked Data (NGSI-LD) [7], promoted by ETSI Industry Specification Group for Context Information Management (ETSI ISG CIM), to define a universal set of standards based on the contextual management of the data. Therefore, adhering the standards and abandoning the code and proprietary technologies can contribute to improving the competitiveness of the agricultural sector. In a world where everything is connected, isolated solutions have no place. By contrast, interoperability must be the cornerstone feature that all solutions adopt, thus contributing to a more productive environment where information is exchanged for a greater good. The proposed solution is a platform that allows for the integration of different suppliers’ devices, and implements standards and open interfaces and data models based on NGSI-LD. Additionally, the platform exploits the stored information to analyze, in real time, the factors associated with the production process, the evolution of the crops, and the optimal use of water for irrigation. The core features the platform are as follows:

1. *Scalability and flexibility*:The platform, instead of being locked to a single provider, is up to date with protocols, technologies, and features that vary rapidly. It is network-independent and can be integrated to work with all vital technological systems;
2. *Interoperable*: The platform offers a wide range of IoT agents that facilitate the connection with devices that use standardized IoT protocols, such as Lightweight Machine to Machine (LWM2M) over Constrained Application Protocol (CoAP), JavaScript Object Notation (JSON), or UltraLight (UL) over HTTP/MQTT, with the possibility of using parameterizing agents to integrate any other type of future protocols. At the application level, the platform is open to integration with other third-party platforms using both API and data model based on NGSI-LD.
3. *Semantic enrichment*: The use of the data model to homogenize the management of the different elements that are part of a farm;
4. *Efficiency and competitiveness*: The model allows precise and timely decisions to be made in terms of management and agricultural processes. The ability to automatically document the health status of the crop or natural resources provides an efficient and effective diagnosis technique for managers.

The remainder of this paper is structured as follows: In Section 2, the proposed solution is introduced, detailing the most unique elements. Section 3 outlines the deployment in a community of irrigators. Section 4 shows the evaluation of the system at different levels of implementation and the results obtained. Finally, in Section 5, the conclusion and new proposals based in these results are described.

### 1.1. Information Models Applied to Agriculture

The data collected by all connected devices are characterized by scalability, heterogeneity, and  dynamism:

- Scalability: Millions of sensors continuously generating large amounts of information;
- Heterogeneity: There is a wide variety of sensors;
- Dynamism: The high speed of generation produced the need to generate data models that allow for better use and dissemination of the information from these devices.

Given the above factors, interoperability mechanisms must be generated that allow for the integration of different devices, applications, or platforms to filter the vast amounts of information that many systems are producing [8]. The shared data models are useful as they are essential resources that improve the communication, knowledge recovery, and interoperability of information systems to develop better applications for the agri-food sector [9].

Today, different data models are available; many of them were developed by the organizations in charge of developing their standards. The Open Geospatial Consortium (OGC) data models are used primarily in geosciences and environmental domains, including the SensorThings API, based on the Observations and Measurements (O&M) data model (OGC/ISO 19156:2011), which transforms the numerous unconnected IoT systems into a fully connected platform where complex tasks can be performed and synchronized. The Open Connectivity Foundation specifies data models based on vertical industries such as the automotive, healthcare, industrial, and smart home sectors. The World Wide Web Consortium Thing Description provides some vocabularies to describe real things but does not focus much on the data. Other vocabularies, such as those offered by the INSPIRE directive [10], define the rules on the interoperability of spatial data sets and their associated services; these are mandatory.

Currently, some resources speed up the data modeling of a specific location or sector. In the field of agriculture, Drury et al. [11] presented different types of tools to help us develop this task:

- Controlled vocabulary (a set of preselected terms or words for a specific domain): An example is AGROVOC vocabulary, promoted by the Food and Agriculture Organization of the United Nations (FAO) and available in multiple languages.
- Miscellaneous ontologies: Crop Ontology, AgroPortal, Dairy Farming Ontology (DFO), AgOnt, CIARD Ring, and Vest.
- Data exchange standards: AgriOpenLink, AgroXML, and AgroRDF; specific ontologies: SSN (for sensor discovery) and Cotton Ontology (diseases and pests that affect cotton).

The basis of many decisions is the uniformity of architectures, and ETSI ISG CIM provides the correct interfaces (006 V1.1.1 (2019-07)) and context information management (CIM) and allows for the unified representation of the information through next generation service interfaces with linked data (NGSI-LD) [12]. This reduces the time and resources required for the management of daily tasks and for the improvement of the productivity and benefits.

A diverse range of ontological models are available for the IoT, developed with different objectives [13,14] that describe various areas, such as Fiesta-IoT ontology, which reuses the results of projects and current EU strategies in semantic web technologies, such as OpenIoT, DUL, VITAL, Spitfire, IoT-O, IoT-A, IoT-Lite, and Sensei. One recent ontology, which is promoted by the ETSI SmartM2M Technical Committee, is the Smart Appliances REFerence (SAREF) ontology, a shared model of consensus that facilitates the matching of existing assets in the smart appliances domain and allows for the separation and recombination of different parts of the ontology according to specific needs. For the agricultural field, the SAREF4AGRI version [15] was created to provide services for animal husbandry, intelligent irrigation, and the integration of multiple data sources to provide support services for decision making.

Some models are promoted by public administrations, such as the standardized water management model applied to irrigation model (MEGA) [16], an initiative of the Ministry of Agriculture of Spain, included in the ISO 21622 standard and managed by TRAGSA Group. The purpose of MEGA is to establish a standardized model that, when applied to irrigation, allows for interoperability between different systems that coexist in the same facility and thus to efficiently manage irrigation water use. The introduction of the standardized model improves how the irrigation schedules and the requirements of the control systems are specified, establishing a clear separation between decision making and execution.

### 1.2. IoT Solutions in the Agricultural Sector

Multiple problems exist in the domain of agriculture, such as irrigation, the application of pesticides and fertilizers, and the monitoring of crops, land, and livestock. Different researchers are working to provide the best possible solutions:

- **Improvements in productivity:** Through the sensorization of crops to provide values in real time, the farmer can apply irrigation, pesticides, and fertilizers only when they are needed. For example, Araby et al. [17] proposed the integration of the IoT and machine learning to predict diseases in horticultural crops before they appear, allowing the farmer to apply the necessary defense mechanisms, thus improving productivity and reducing the use of pesticides. Trilles et al. [18] presented a low-cost sensor-equipped platform, SEenviro, which applies a disease model for alert management in vineyards;
- **Detection of undetected problems:** Using satellite images or drones, harmful agronomic factors, previously untreated, can be detected and mapped in crops using remote-sensing techniques. For example, De Rango et al. [19] monitored crops using the images provided by a drone to see if they had parasites and to decide if subsequent treatment was necessary, representing a new technique for the coordination and control of the drone fleet in precision agriculture (PA);
- **Monitoring the behavior of plants:** Through the use of artificial intelligence to analyze the entire dataset obtained from crops to make future predictions, the PLANTAE platform [20] is a system capable of managing the agricultural process and simultaneously using machine-learning techniques to detect possible diseases in plants. Other works, such as that of Choudhury et al. [21], monitored the behavior of plants to avoid pests and diseases. Through the use of a mobile applications, farmers report events that improve the models of diseases used;
- **Efficient water management:** Crop water adjustments should avoid water stress. Riquelme et al. [22] showed how the use of cloud services involving the FIWARE platform allows for the improvement of the management of water used for irrigation in areas with water deficits. Another project to be considered, also based on FIWARE, is the SWAMP platform [23]. Its primary objective is to develop innovative methods based on the IoT for the intelligent management of irrigation water, using the semantic characteristics provided by a context engine based on the SPARQL Protocol and RDF Query Language (SPARQL) Event Processing Architecture;
- **Improvement in greenhouse management:** By monitoring its different components, Zamora- Izquierdo et al. [24] proposed a flexible platform capable of meeting the needs of hydroponic crops in a greenhouse with complete recirculation. For this FIWARE-based deployment, Message Queuing Telemetry Transport (MQTT) communications were used, with NGSIs being used as a means to represent the information. Somov et al. [25] constructed a system to monitor both the conditions established in a greenhouse and the behavior of plants for the prediction of the growth rate of tomatoes in different environments.

IoT platforms can be considered the backbone of any industrial sector that wants to provide its activity with a smart component. These platforms collect and store data in a distributed database for the filtering, analysis, calculation, decision making, management, translation, and visualization of data in new services. Several analyses of the different types of platforms for the integration of IoT services and their characteristics can be found, such as that provided by Silva et al. [26], although a results comparison is difficult because of the lack of standardization.

## 2. Proposed System

This article proposes an open and interoperable platform for irrigation community management. The platform, based on standard and open interfaces and protocols, allows for the integration of heterogeneous information sources as well as interoperability with other third-party solutions for exchanging and exploiting this information. Additionally, this platform exploits the stored information to analyze, in real time, the factors associated with the production process, the evolution of crops, and the optimal use of water for irrigation.

### 2.1. Proposed Data Model

As a result of the lack of the compatibility necessary to manage the large amount of data generated by the agricultural sector, agricultural managers must be provided with standardized information models that permit farmers to make the best possible decisions, allowing them to take advantage of the available data and knowledge. Therefore, a data model is proposed here that fits the functional requirements of the users.

The proposed data model is based on interviews with farmers, responsible technicians, and farm managers about the useful and necessary information that should be managed on a farm. The existing models are too specialized; although they cover the information that needs to be managed, their implementation is time-consuming. Therefore, we decided to develop a new model that would meet the needs of our users.

**Figure 1:**
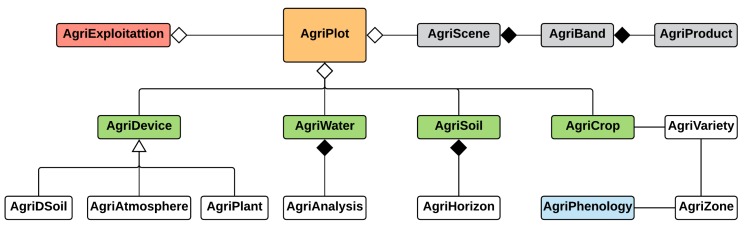
Specific data model for agricultural plot management.

Conceptually, the model presented is based on the proposed by FIWARE harmonized data models [27], specifically, the AgriFood model that includes requirements for irrigation and crop control. The model was constructed as an agile tool to respond to the requirements farmers allowing them to characterize the plots, reduce the hydric needs of the crops, and control the soil and water quality and the associated atmospheric conditions. This first version of the model focuses on the efficient management of the crop plots and all the included elements, as can be seen in the class diagram in Figure 1.

The proposed data model focuses on the management of crop plots represented by the *AgriPlot* entity, which is part of an agricultural holding, represented by the *AgriExploitation* entity. The plot is characterized by four pillars that allow it to be uniquely defined:

- **Devices**, represented by *AgriDevice*, are the devices that provide information to the plot and are classified based on the parameters studied: *AgriDSoil*, those that obtain soil values (soil moisture probes, temperature and soil dissolution); *AgriPlant*, those that measure the evolution of the crop (leaf/trunk diameter, stem water potential, and dendrometers); and *AgriAtmosphere*, which are the devices that record the atmospheric conditions (temperature, humidity, radiation, and wind speed). These data are used to optimize agricultural decisions;
- **Water**, represented by *AgriWater*, indicates the type of water used and the parameters that affect irrigation. *AgriAnalysis* manages the water analysis conducted at different points of the water distribution network of the farm. These analyses are usually necessary for fertilization and irrigation, since they describe the quality and quantity of nutrients carried by the water;
- **Soil**, represented by *AgriSoil*, indicates the type of soil present in the plot. This entity can be more detailed by indicating the *AgriHorizon*, which includes the different characteristics of the layers (horizons) that define the ground;
- **Cultivation**, represented by *AgriCrop*, defines the crop and the variety, *AgriVariety*, with which the plot is associated. The crop is determined by the different phenological phases that determine its growth; *AgriPhenology* provides the crop coefficients and their duration over time. The phenology is determined by the variety of the crop and the climatic zone, *AgriZone* since the zone influences the optimal climatic conditions for the development of the crops.

Another feature to be considered is the **Aerial Images** management, represented by the *AgriScene* entity, including images or scenes from any satellite or drone; these scenes are composed of bands, *AgriBand*, of different wavelengths. In turn, these bands can generate derivative products, *AgriProduct*, after processing, such as mosaics (adding two or more adjacent scenes) or indices of vegetation or water, useful for detecting problems in the fields.

The main feature of the IoT-based interoperability mechanisms is the possibility of exchanging information homogeneously in different systems or applications. One of the elements that enables this continuous exchange of information is the use of data models. To acquire more significant value, the specific vocabulary of the sector must be unified, which is defined by the set of attributes that compose the entities and their relationships [28]. As NGSI-LD allows us to link data from other vocabularies or ontologies, we added a level of interoperability to the model. For these reasons, the use of information models is considered beneficial to allow for the integration of any device or characteristic element of the study sector.

In Section 3.2, an instantiation of this model is provided using NGSI-LD. The advantages of the use of NGSI-LD concerning NGSI are as follows:

- The information model is based on graphs and focuses on information. The concept of *Relation* appears. Entities can have properties and relations. Instances of each of the entities can be the object of the properties or relationships;
- All data types in NGSI-LD can be associated with unique Uniform Resource Identifier (URI) corresponding to well-established semantic identifiers;
- It allows one to make references to vocabularies: all terms are defined unequivocally. This allows users to refer to their information definitions;
- The model and query language is more constrained;
- The use of JSON-LD allows us to operate with linked data to unify vocabularies;
- There were syntactic differences: the metadata dictionary is no longer needed, GeoProperty is used instead of geo:json, JSON-LD @context is included, TemporalProperty is used instead of DateTime, and an “object” field is used to encode the relation target.

### 2.2. System Architecture

Since the new IoT devices and the new communications technologies provide the user with faster and more efficient communication, we propose an affordable platform, independent of the provider and interoperable, as the cornerstone for all connections between applications/services and the irrigation elements. In this way, we offer a common framework so that applications can interact with any aspect that is part of a farm in a similar way, while being able to act on any device or controller using the same set of instructions, thus allowing for greater interoperability between different manufacturers or services.

**Figure 2:**
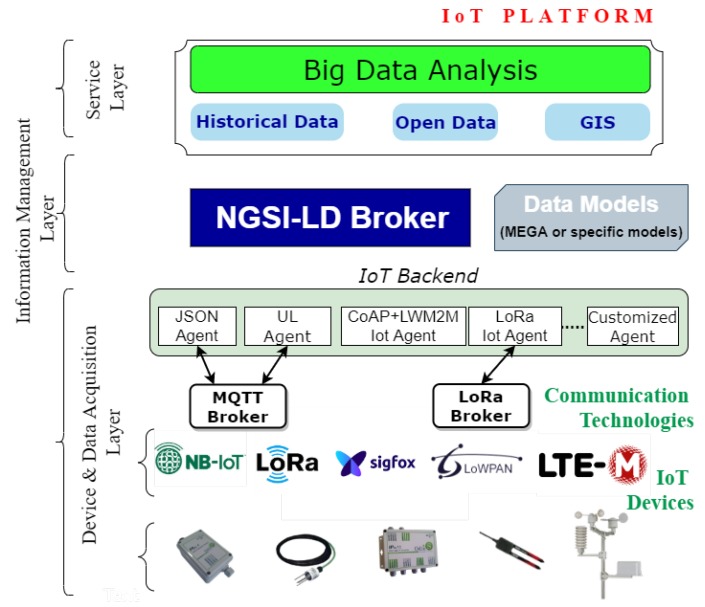
General vision of the proposed architecture.

The architecture of the platform proposed in this paper is depicted in Figure 2. It has a layered modular form ranging from the deployment of sensors and the monitoring of techniques for data extraction to the intelligent processing of data. Each of these layers is based on open and standard initiatives, such as the one provided by the FIWARE community [29] or the ETSI ISG CIM group.

The first layer, *Device and Data Acquisition*, represents the different sources of information that form the system; these include sensors, actuators, open information available on the Internet, and databases with updated information of interest. These devices employ different communication technologies to transmit information to an IoT Backend module formed by IoT Agents. This module acts as an intermediary with the second layer and transforms information unidirectionally or bidirectionally (in the case of actuators), allowing the IoT devices to interact with the platform.

A specific module is required because of the restrictions imposed by the modules on the processing capacity, memory, and even the available power to these devices. These restrictions usually prevent them from using heavy protocols at the application level, so they use lightweight protocols such as CoAP or MQTT or integrate other solutions such as LoRa and Sigfox to perform communications with the different devices through Ethernet or mobile networks (e.g., GPRS/3G/4G/NB-IoT/5G). As such, the IoT backend module component acts as an intermediary by translating the information sent through these protocols to the interface made available by the broker through NGSI-LD.

**Figure 3:**
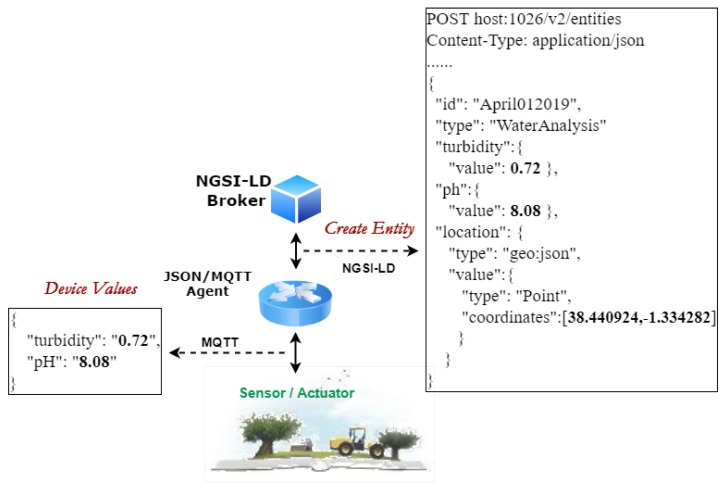
Data model from Internet of Things (IoT) level to applications.

The second layer, *Information Management*, contains software components in charge of data storage, processing, and distribution. The distributed infrastructure is composed of servers in the cloud that work together to manage massive amounts of data and make them available to the upper layer. The NGSI-LD interface is used to send data updates and receive notifications about data changes. Figure 3 presents an example of how the information provided by an IoT gateway, which implements JSON over MQTT, is integrated into the platform.

The element chosen was the device that analyses the water at the exit of the wastewater treatment plant and determines the levels of *turbidity* and *ph* of the water. Once the entity is created in the broker using the NGSI-LD data model, consumers can retrieve this information following two different approaches: query or subscription.

The core of this platform is the NGSI-LD broker. It is an information broker that exposes an HTTP REST API based on NGSI-LD for both registration and consultation, as well as a subscription/ notification approach. This is the second point where interoperability is a significant aspect. The use of NGSI-LD provides various possible interactions with third-party platforms in both directions. One of these solutions that has been integrated into the platform through the development of a connector is MEGA [16]. The MEGA standard (ISO 21622) provides guidelines for the implementation of a standardized model applied to irrigation that improves the specification of irrigation schedules and the control of the system requirements. Within this management layer, the stored information of each entity is overwritten, with only the current state being kept; the information is stored in a nonrelational database, MongoDB. This database contains data referring to infrastructure and information flow, as well as information about users and organizations. All this information is modeled using data models that allow for the unification of data structures using standards and also ensure the generality necessary for the subsequent extensibility.

The last layer, *Service*, serves as an interface between users and the central layer to offer different solutions to the problems that are generated in an agricultural operation, such as water management, irrigation planning, data analytics, and monitoring of environmental parameters. The incorporation of other modules allows us to provide our solution with more capabilities due to the possibility of integrating new components through connectors with NGSI-LD. The NGSI-LD interface is used to send data updates and receive notifications about data changes. A change in the configuration parameters triggers control actions that are managed by subsystems at the user level. As a result of the use of specific connectors, the information is consulted or notified to higher modules that are in charge of propagating it for other interests, such as historical information, large-scale data management, or integration with geographic information systems (GIS). For this purpose, a representational state transfer interface (REST) is used, which applies NGSI-LD for communication between the final applications and the analysis modules.

## 3. Case Study in an Irrigation Community

This section validates our data model and the architecture of the platform. It was implemented in an irrigation community where different systems, such as supervisory control and data acquisition (SCADA), GIS systems, and sensors from different manufacturers, were integrated by the platform.

### 3.1. Scenario

Irrigation communities are corporations attached to basin organizations, which are responsible for the management of the shared use of public common water. To this end, they relied on automatons and specific irrigation systems for years, which were tightly coupled, allowing for full interaction between the devices and the software, but avoiding any other possibility of interacting with third-party devices and software applications.

The Miraflores irrigation community is located in the municipality of Jumilla (Murcia) [30] in the Segura river basin and has almost 1000 community members. The area comprises about 1330 ha of agricultural land, mostly devoted to woody crops and irrigated by localized irrigation. The main crops produced are fruit trees, especially pear, peach, and apricot. The community uses surface water, 3.8 ${\mathrm{hm}}^{3}$ annually, and water from the Jumilla sewage plant, 1.5 ${\mathrm{hm}}^{3}$ annually. This treatment plant delivers the reclaimed water at its exit, from where the community drives the available flows to six interconnected regulation rafts, with a total capacity of 1 ${\mathrm{hm}}^{3}$. The distribution network conducts water from the rafts to the various irrigated farms, equipped with automatic flow control counters and setpoints, ensuring an average allocation of 4025 $m^{3}/\mathrm{ha}/\mathrm{year}/\mathrm{farm}$.

One of the main problems to be solved is the integration of the different technological elements (SCADA, drawing software, independent sensors, and third-party services) that provide information to the community in a single control point. The data from SCADA that are integrated into the platform are shown in Table 1. The results derived from the water analysis conducted at least twice a month were also integrated into the platform.

### 3.2. Equipment and Implementation

**Figure 4:**
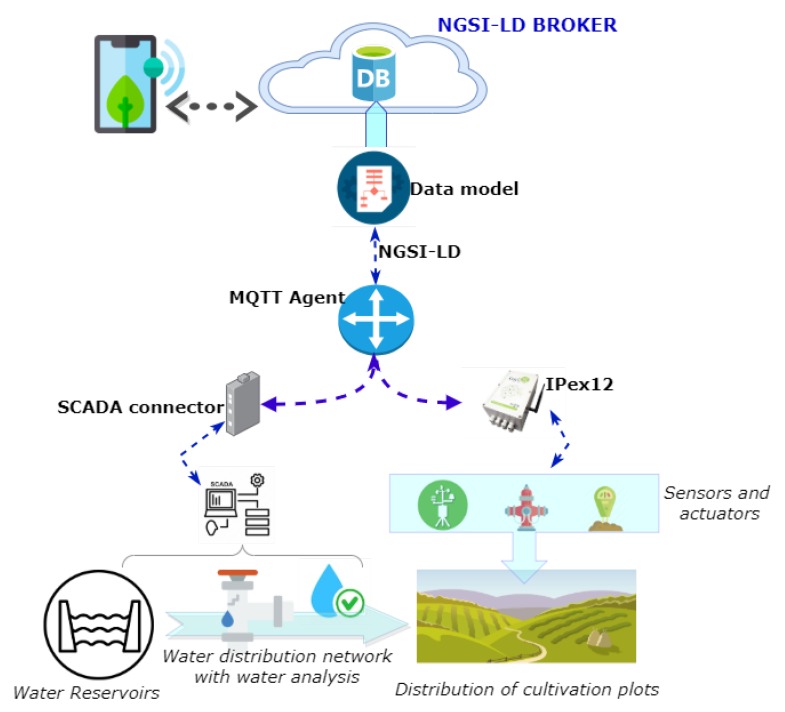
Layout of the case study.

Figure 4 shows the general scheme that was followed to address the deployment of the platform. The first step was to channel water from the headwater reservoir, with its corresponding water analyses, to the agricultural plots. The plot is one of the key elements of the system. All the devices needed for its definition are integrated using dataloggers and IoT connectors. Through this process, SCADA data and the sensors that interact with the plot (soil moisture sensors, agricultural weather station, and hydrants) are integrated into the systems. The information is transferred to the data model for later visualization (using mobile or web applications) and analysis by the users of the irrigation community.

The deployment involved the installation of controllers for monitoring, and remote control in outdoor environments were provided by Odin Solutions [31]. The cost of implementing the platform was reduced due to the open source feature of the platform. This feature decreased the budget needed for dataloggers managing different devices or sensors in the platform. In this instance, the cost of the chosen datalogger, *IPex12*, has an average price of around 600 euros (including configuration and installation) and varies according to the number of sensors integrated into the system. The main characteristics of *IPex12* are: 32-bit CPU and 4 MB of memory expandable with microSD, Ethernet, USB, CAN, 3xRS232, 1xRS485, and 12 I/O ports, which can be configured by software as digital or analogue input/outputs. It supports Third-generation Networks (3G), IPv6 over Low power Wireless Personal Area Networks (6LowPAN), Sigfox, Narrow Band IoT (NB IoT), and LoRA. Each *IPex12* is able to manage eight additional slaves I/O boards using CAN. The controllers are configured effortlessly through their web server, which permits us to configure tasks independently from the MQTT commands received from the cloud computing layer. IPex12 is specially designed for outdoor settings such as the agricultural scenarios, since it is provided with water- and dust-proof enclosure. It is provided with a battery and a solar panel.

The dataloggers have the ability to monitor sensor readings of soil conductivity, soil moisture, soil temperature, water meter readings, and meteorological parameters, among others, and to operate the solenoid valves to control the irrigation system.

The cloud server is a PowerEdge R7515 Server, with an AMD EPYC 7402 2.80 GHz, 32 GB of RAM, and two solid state drives (SSD) of 480 GB each. It also runs the Ubuntu 18.04 LTS Server and LibVirt/KVM. A single virtual machine was used here for the global broker, data saving, big data processes, and to host management with web services. The virtual machine uses 28 GB of memory and 400 GB of hard disk capacity. A good performance is possible at the moment, but given the flexibility of our architecture, more resources could be added by modifying or moving virtual images.

Once the different modules that form the platform were configured, the entities were defined in the broker so that their subscriptions could be made later. The broker manages subscriptions to the creation or changes of entities. All these changes are stored in MongoDB, a nonrelational JSON-LD object-based database, to provide fast and flexible access to information. The speed of processing is fundamental because, otherwise, a bottleneck is created that would slow down the rest of the  implementations.

To improve the data of the irrigation plots, the data model proposed in Section 2.1 was used according to the needs proposed by the users of the irrigation community. A model instantiation was constructed through NGSI-LD of an apricot tree plot where a soil moisture sensor was installed. The soil moisture sensor is listed in Listing 1 and the crop is defined in Listing 2.

**Listing 1:**
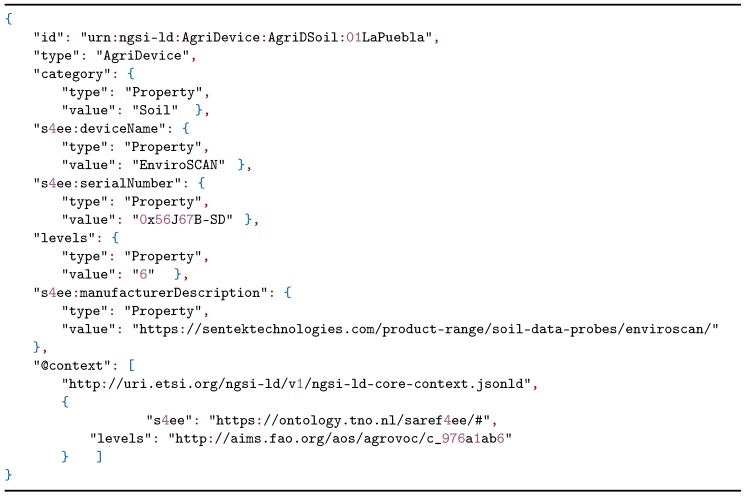
Next generation service interfaces with linked data (NGSI-LD) example representing an AgriDevice.

**Listing 2:**
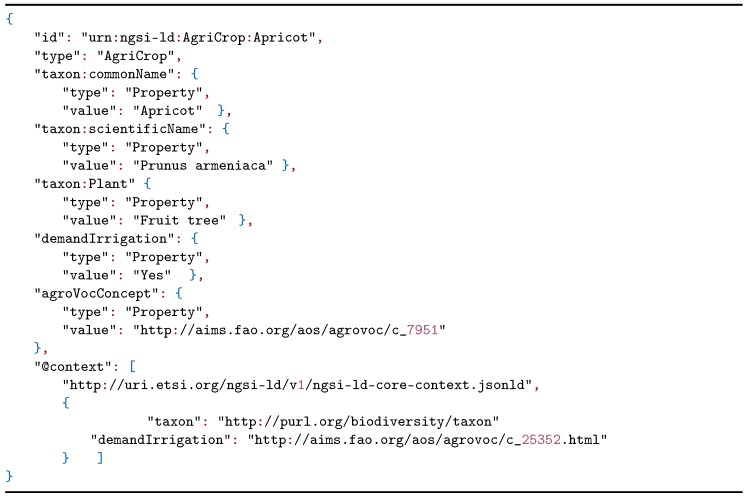
NGSI-LD example representing an AgriCrop.

After defining the crop sown in the plot and the devices that help to improve irrigation management to improve production, we detailed the characteristics that define the plot. In Listing 3, the parameters necessary for the calculation of the water needs of the crop were observed, including plantation frame (*density*), emitter flow (*flowRate*), number of emitters (*emitters*), and tree crown diameter (*diameter*).

**Listing 3:**
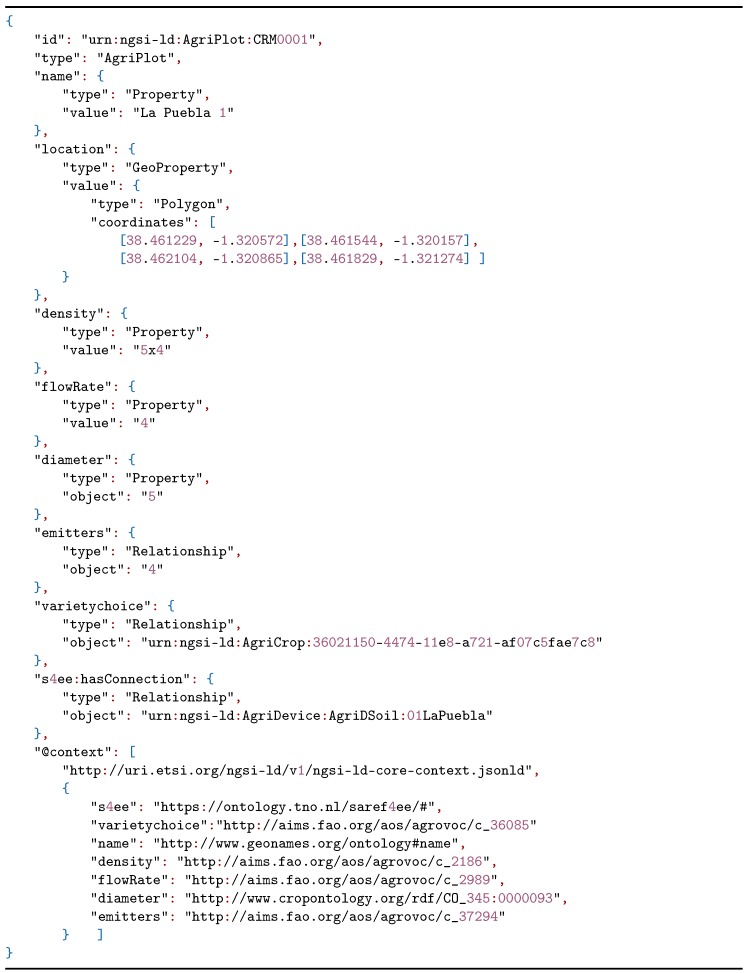
NGSI-LD example representing an AgriPlot with related entities.

## 4. Evaluation and Validation

This section describes the validation of the platform in a community of irrigators from three different perspectives. The tests were conducted under a controlled environment with the equipment described in Section 3.2.

### 4.1. Information Broker Evaluation

The main element of the platform is a NGSI-LD broker, which is responsible for storing all the information as well as processing the different queries, answers, subscriptions, and the management of the information providers. For this reason, an analysis was performed by selecting the following metrics: CPU usage, memory consumed, and response times, according to the operations performed.

**Figure 5:**
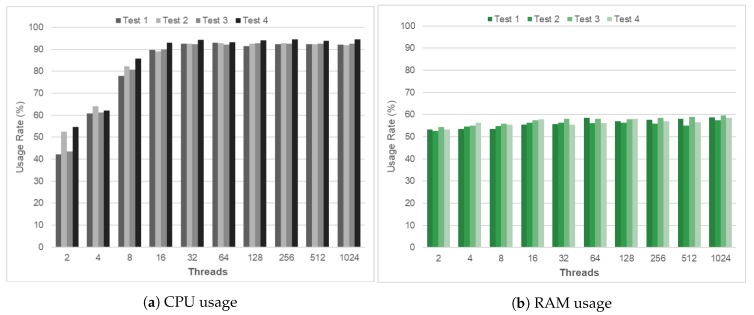
Evaluation of platform scalability.

Firstly, different execution tests were conducted to check the platform ability to perform the changes without losing the quality of service. The tests were performed with the same type of requests, checking their response time in terms of both CPU and RAM. All tests were based on a one-minute execution varying the number of simultaneous communications or connections with which it was performed. Figure 5 shows the results of the tests of the behavior of the processor and the memory.

Figure 5a shows that the CPU requirements increased by around 20% in the first intervals (2, 4, and 8 threads). From this moment on and up to 1024 threads, the CPU remained stable with average values close to 90%. Figure 5b shows that the behavior in the use of the RAM remains practically stable from the beginning to the end without altering the consumed resources. The increase in the use of the RAM during the tests performed varied between 5% and 9%, values that are considered optimal.

**Figure 6:**
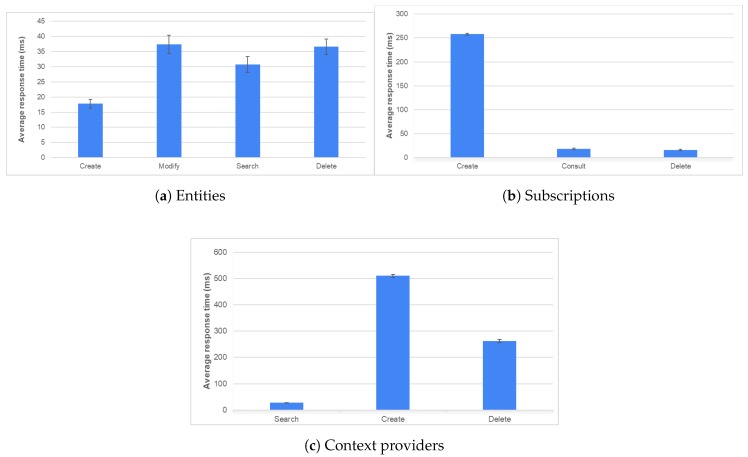
Response time for operations with different operating methods.

The behaviour of the platform was evaluated based on the information distribution characteristic that the broker had acquired. To this end, the response time obtained after performing various operations on the different broker operating modes is analyzed; Table 2 shows the most significant values obtained in the tests. The graphs shown in Figure 6 show the times for the different modes: entity management, subscription management, and context provider control.

- The publication/subscription model enables the reduction of the number of queries because it is not necessary to make periodic queries to receive updates to the data stored on the platform. The creation time is longer since it has to be adjusted to the proposed data model;
- The management of external context suppliers is a process that speeds up access to the information, making the process more transparent for the final client. The broker’s mission is to act as a proxy between the client and the context provider. For this reason, search and consultation times are shorter compared with the rest of the operations.

### 4.2. Evaluation of Platform Usage

**Figure 7:**
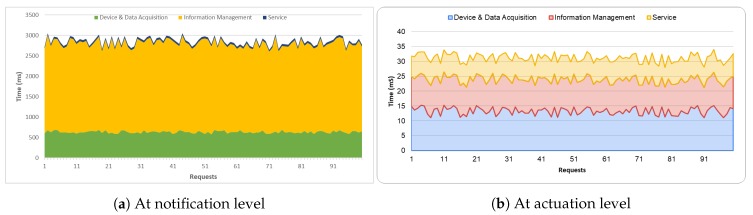
Response time according to the layers that form the platform.

This section provides an evaluation of the response times of the platform based on the three layers that form its architecture. Figure 7 shows the times obtained for the system after the execution of 100 requests. Figure 7a shows the notification times at its two levels: at MQTT level (MQTT broker notifies the IoT Agent) and at NGSI-LD broker level. The times of the *Information Management Layer* are longer, with an average of 21.69 s, since all the semantic enrichment provided by the use of the data model is performed in the database (MongoDB). However, in exchange for this increase in time, it is possible to have all the relevant information of the irrigation community homogenized and integrated into a single point. Figure 7b shows the system’s capabilities in terms of performance management. The graph shows that if the device is connected, the response time is less than if it is not; the average response time of 31.18 ms is considered low.

### 4.3. Use of the Platform at the Agricultural Level

Data interoperability is of critical importance. Here, the proposed solution (data model and platform) provides the necessary data translation mechanisms by combining the use of a data model together with standardized solutions, such as those provided through NGSI-LD. This proposal enables the integration of multiple systems/devices or data sources, resulting in an open and interoperable data integration model according to the changing requirements of farms. The use of data models contributes to obtaining performance indicators, decision making, and sharing data with different farms.

**Figure 8:**
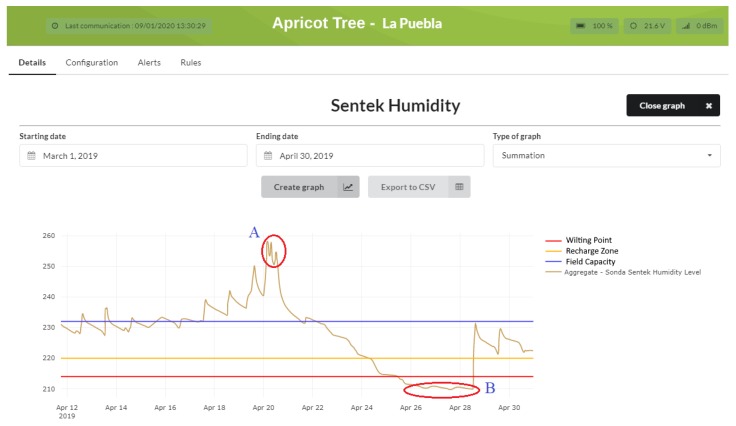
Evolution of the humidity of an apricot crop based on set water levels.

Figure 8 shows the evolution of irrigation before the platform was installed. The analysis of the data revealed the irregularity of the irrigation system. To have a continuous and homogeneous irrigation system, we deemed it necessary to define irrigation thresholds provided by the agricultural technicians. As the *field capacity* and the *wilt point* determine the maximum and minimum limits of the soil humidity that can be used by the crops, we concluded that the amount of water between these two values is the functional water or humidity available to the plant. Once the community technicians defined the limits for the crops, tests were conducted on an apricot tree plot to evaluate the irrigation efficiency: the first as soon as the platform was deployed (between March and April), and another after improving the irrigation programming based on the results of the first test (between June and July).

Once the first test was analyzed, Figure 8, we found that during irrigation, two critical zones were generated, A and B, with extreme peaks that were outside the recommended limits, indicating that water use was not efficient, thus a scarce resource was being wasted. Zone A indicates that root asphyxiation was produced (a limitation of the capacity of the plants to breathe through the roots) in the crop by excess water; Zone B indicates that the plant does not have ability to supply itself with water, consequently reducing the quality of the production of the crop. Once the maximum and minimum thresholds are set, rules can be defined for efficient irrigation. When the thresholds are exceeded or not reached, the platform generates notifications to users through the mobile application. These rules can also be associated with the activation of digital inputs for the start-up of any device that improves irrigation efficiency.

**Figure 9:**
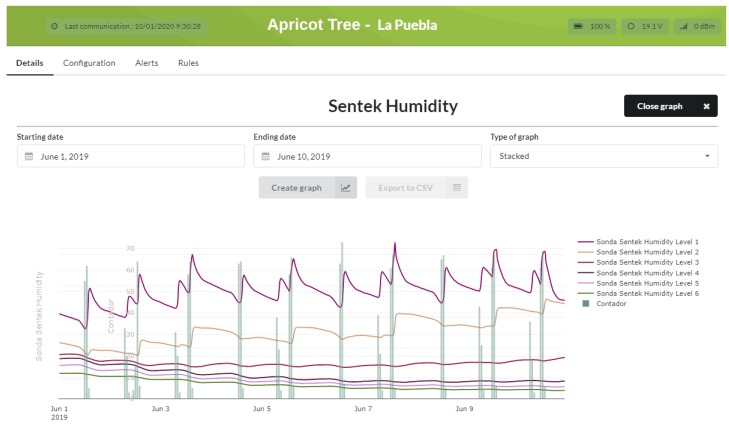
Evolution of efficient irrigation, comparing soil moisture and water contribution by the hydrant.

As a result of the use of the platform and the analysis of the data obtained, and with the help of an agricultural technician, the relevant irrigation limits are established or adjusted. In addition, other useful parameters can be established, such as the root network, the type of soil, or the disposition of the probes. Once the irrigation programming was changed, Figure 9 shows that the irrigation was efficiently applied. The water contributions and vertical column are in line with the humidity curves, showing stable and uniform sharing. By controlling the hydrants and applying filters based on the limits set, irrigation was controlled to maintain the humidity within the desired parameters, or to inform of possible alerts to activate programmed irrigation to avoid a reduction in production.

The versatility of the platform is stressed by the ability to export selected data for further processing or study, as shown in Figure 8 and Figure 9 using the *export to CSV* option.

## 5. Conclusions

This work demonstrated the importance of interoperability in the field of agriculture, water management, and irrigation systems. Herein, an interoperable and open platform is presented which is capable of integrating heterogeneous data sources at the IoT level to aid and improve the decision making in a community of irrigators. The platform enables the integration of controllers and sensors from different manufacturers, and thus represents a unique access point for all this information. As such, improving the techniques used in the agricultural sector is possible, thereby sustainably obtaining higher economic, environmental, and social yields. As a result of its interoperable nature, the platform can be combined with other systems to expand its range of services. The interoperability aspect was tested in a real-world context where successful integration with other systems already deployed in the facilities of the irrigation community was possible thanks to this characteristic. The platform integrates different protocols at the IoT level, such as MQTT, CoAP, LoRa, SigFox, and HTTP. They transport the information following different data formats. Nevertheless, a set of them promote interoperability, such as Lightweight Machine to Machine (LWM2M), JSON, and UltraLight. These representation formats were adopted by the presented platform as to facilitate its integration into IoT devices, making use of this technology. The agents of the IoT backend of our platform perform the adaptation of this information to the NGSI-LD interface and data model. After analyzing the obtained results in the community of irrigators, the following conclusions were obtained:

1. A homogeneous data model was proposed that meets the specific needs of agriculture, such as efficient water management. This model was validated on the platform using an NGSI-LD broker. The application of this model in an irrigation community provides its managers with the capacity to manage the agronomic information and its relationship with the devices that provide information to improve irrigation water;
2. The platform was validated using metrics to check its behavior. Firstly, the scalability of the main component, the NGSI-LD broker, was analyzed based on the average use of the CPU (83.21%) and memory (56.39%). At the latency level, measures were recorded on the most relevant operations of the broker, highlighting among them the average time, 510.28 ms, of the creation of a context provider. The platform was validated as a whole, showing the time delay from the moment the device receives the information and when it is received by the platform (2.97 s); and from the moment the action is performed (31.17 ms);
3. At the user level, the platform was validated in several apricot tree plots, improving the management of the water used. This improvement was achieved because of the definition of optimal irrigation thresholds for each crop and by generating filter notifications that, under certain conditions, allow the hydrants to be adjusted, thus enabling efficient water use.

In the future, the speed of computing and latency should be compared by replicating the platform in edge computing with current cloud computing to detect which one provides better performance and response times. One of these involves the processing of satellite images. At the plot level, this feature will monitor the main characteristics of the crop with applications based on remote sensors. This new attribute will focus on the diagnosis, management, and control of irrigation. Currently, this feature uses images of the Sentinel 2 satellite, which is more focused on agricultural issues, but soon the PAZ satellite (the first Spanish radar earth observation satellite, which is included within the National Earth Observation Programme) will be used. Another future line of research is the development of a data analysis module that will allow us to cross check and analyze the history of the data acquired. This will generate better results in agricultural management, increase productivity, and reduce expenses, such as by reducing the consumption of water, nutrients, and phytosanitary products.

## Figures and Tables

**Table 1:** Type of facilities from supervisory control and data acquisition (SCADA) that are integrated into the platform and the associated variables.

Facilities	Quantity	Sensors
Header reservoir	1	Reservoir level, filtered outlet pressure and reservoir inlet, ph, turbidity
		ammonium, nitrate, conductivity, phosphates, potassium, chlorides
Reservoirs	6	Reservoir level
Wells	8	Water temperature and flow, pressure, and level deepwater
Filters	7	Inlet and outlet pressure, cleaning flow, output flow
WWTP	1	Network and solar pumping flow, network and solar pumping pressure
		wind speed, radiation

**Table 2:** Average response times (ms) for each type of operation and mode.

Operating Modes	Create	Modify	Search	Consult	Delete
**Entity**					
*Mean Value*	17.76	37.31	30.72	-	36.56
*Confidence Interval*	1.46	2.99	2.63	-	2.56
**Suscription**					
*Mean Value*	257.95	-	-	18.57	16.18
*Confidence Interval*	2.23	-	-	1.54	1.56
**Context Provider**					
*Mean Value*	510.28	-	27.51	-	262.45
*Confidence Interval*	5.25	-	1.37	-	6.39
